# Cell Signals Influencing Hepatic Fibrosis

**DOI:** 10.1155/2012/158547

**Published:** 2012-08-29

**Authors:** Min Cong, Keiko Iwaisako, Chunyan Jiang, Tatiana Kisseleva

**Affiliations:** Department of Medicine, School of Medicine, University of California, San Diego, La Jolla, CA 92093, USA

## Abstract

Liver fibrosis is the result of the entire organism responding to a chronic injury. Every cell type in the liver contributes to the fibrosis. This paper first discusses key intracellular signaling pathways that are induced during liver fibrosis. The paper then examines the effects of these signaling pathways on the major cell types in the liver. This will provide insights into the molecular pathophysiology of liver fibrosis and should identify therapeutic targets.

## 1. Introduction

Fibrosis is the outcome of many chronic liver diseases [1], including hepatitis B virus (HBV), hepatitis C virus (HCV), alcoholic liver disease, and nonalcoholic steatohepatitis (NASH), liver intoxication (drug or nutritionally related) [1]. It is manifested by massive accumulation of the extracellular matrix (ECM) and scar formation. Several injury-triggering events play a critical role in the pathogenesis of liver fibrosis. Chronic liver injury damages the endothelial barrier and induces apoptosis of hepatocytes. Apoptotic bodies and necrotic cells release chemokines that recruit inflammatory cells to the injured liver and release fibrogenic and inflammatory cytokines (TGF-*β*1, IL-6, IL-1*β*, and TNF-*α*) that activate macrophages and hepatic stellate cells [2]. BM-derived and liver resident macrophages (Kupffer cells) are believed to be the major source of TGF-*β*1 in fibrotic liver [1, 3]. TGF-*β*1 is critical for the activation of fibrogenic myofibroblasts, which in response to injury upregulate *α*-smooth muscle actin (*α*-SMA) and secrete extracellular matrix proteins, mostly collagen Type I (Col), I, and III [3, 4]. Hepatic stellate cells (HSCs) contribute >80% of the myofibroblasts in the fibrotic liver in response to an hepatotoxic injury [5]. HSCs express unique markers such as Desmin and glial fibrillar acidic protein (GFAP), which distinguish them from other cells in the liver [1]. Under physiological conditions, HSCs store Vitamin A and retain a quiescent phenotype (qHSCs), but following TGF-*β*1 stimulation, PDGF, or matrix stiffness, or other fibrogenic stimuli they rapidly activate into type I collagen, *α*-smooth muscle actin expressing myofibroblasts (aHSCs) [1, 4, 6]. In addition to HSCs, cholestatic liver injury causes activation of portal fibroblasts, which differentiate into myofibroblasts and contribute to scar formation [7, 8]. Cholestatic injury also triggers cholangiocyte activation, and proliferation of the bile ducts (ductular reaction) [1].

## 2. Overview of the Signaling Pathways Critically Involved in Pathogenesis of Liver Fibrosis

### 2.1. TGF-*β*-Smad2/3

Signaling by the cytokine transforming growth factor-*β*1 (TGF-*β*1) plays a pivotal role in growth and differentiation, maintenance of liver homeostasis, terminal differentiation of hepatocytes and other epithelial cells, and cytokine-mediated mitogenic signaling [1, 9, 10]. The TGF-*β* superfamily is composed of many multifunctional cytokines, including TGF-*β*s 1, 2, and 3, activin, and bone morphogenic proteins (BMPs) [11, 12]. Under physiological conditions TGF-*β*1 regulates tissues remodeling and apoptosis to maintain cellular homeostasis [13, 14]. Under pathological conditions, TGF-*β*1 is the strongest known inducer of fibrosis, being a direct regulator of fibrillar collagens, TIMP1, plasminogen activator inhibitor 1 (PAI1), p300 [15]. In response to injury, TGF-*β*1 orchestrates a cross talk between parenchymal, inflammatory, and myofibroblast cells. Although many cells in the liver may produce TGF-*β*1, Kupffer cells and recruited macrophages are the major source of TGF-*β*1 in the fibrotic liver. TGF-*β*1 is critical for activation of HSCs into myofibroblasts [1]. aHSCs, and to lesser extend sinusoidal endothelial cells (ECs), also contribute to TGF-*β*1 production [1].

To mediate its function, TGF-*β*1 undergoes several important posttranslational modifications. TGF-*β*1 is synthesized as a nonactive proform, cleaved intracellularly by the endopeptidase furin to generate mature form, but remains biologically inactive due to its association with a complex of two proteins: latency-associated peptide (LAP) and latent TGF-*β*-binding protein (LTBP). This large TGF-*β*1-associated complex is then secreted into the ECM, where it is crosslinked by tissue transglutaminase and stored as a reservoir without any effect on the surrounding tissue [16]. Inactive TGF-*β*1 interacts with fibronectin. Briefly, two variations of FN exist: plasma FN (pFN), a dimeric and soluble form secreted by hepatocytes directly into the circulation; cellular FN (cFN), found in the ECM of tissues in a multimeric form containing alternatively spliced variants of extra domains EDA and EDB [17]. EDA cFN secreted by the cells or already present in the ECM activates latent TGF*β* [17]. Mature TGF-*β*1 is released from LAP/LTBP, the latency maintaining protein complex, by activation of thrombospondin 1 (TSP-1) [18, 19], *α*v*β*6 and *α*v*β*8 integrins (heterodimeric matrix receptor expressed by epithelial cells, some dendritic cells, and macrophages), or acidification [16, 18, 20–25]. Alternatively, TGF-*β*1 can be activated by several proteases such as plasmin or matrix metalloproteinases MMP-2 and 9, which directly induce degradation of the LAP/LTBP complex [26]. Neutrophil elastase, a serine protease released by neutrophil degranulation, has also been implicated in activation of latent TGF-*β*1 [24, 25, 27].

**Figure 1:**
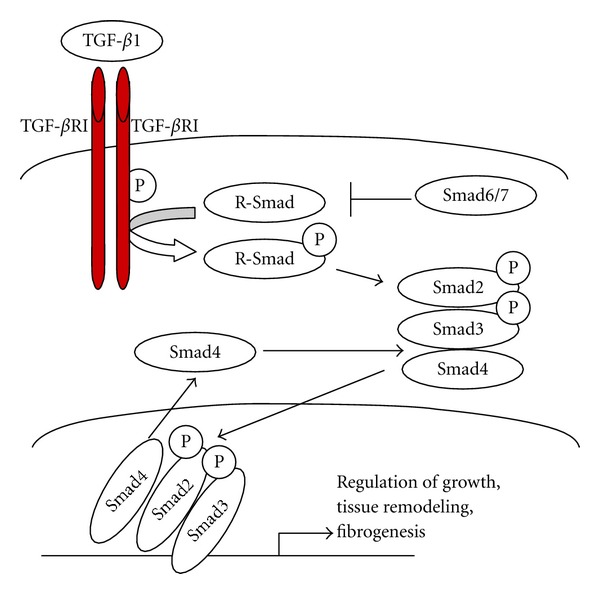
TGF-*β*1 signaling. At the cell surface, TGF-*β*1 binds a complex of transmembrane receptor serine/threonine kinases types I and II (TGF-*β*RI and TGF-*β*RII) and induces transphosphorylation of the the type I receptor by the type II receptor kinases. The activated type I receptor phosphorylates Smad2 and Smad3, which then form a complex with a common Smad4. Activated Smad complexes translocate to the nucleus and function as transcription factors. Activation of R-Smads by type I receptor kinases is inhibited by Smad6 or Smad7. R-Smads and Smad4 shuttle between nucleus and cytoplasm.

TGF-*β* mediates its biological function via signaling through the downstream molecules Smads (Figure 1). The Smad family of proteins contain a conserved Mad-homology (MH) 1 domain, an intermediate linker, and a MH2 domain [28]. There are three classes of Smads: (1) receptor-regulated Smads (R-Smads), which include Smad1, 2, 3, 5, and 8; (2) common-mediator (co-Smad) Smad4; (3) antagonistic or inhibitory Smads, Smad6 and 7 [10, 29]. Smads regulate the signals from the receptors for TGF-*β* superfamily members to the nucleus. Catalytically active TGF-*β* type I receptor (T*β*RI) and activin type I receptor (ActRI) phosphorylate serine residues of receptor-activated Smad2 and Smad3 [30]. Smad proteins have intermediate linker regions between conserved Mad homology (MH) 1 and MH2 domains. TGF-*β* Type I receptors differentially phosphorylate Smad2 and Smad3 to create C-terminally (C), linker (L), or dually (L/C) phosphorylated (p) isoforms. Although COOH-tail phosphorylation by T*β*RI is a key event in R-Smad activation, additional phosphorylation can positively and negatively regulate R-Smads pathway. Thus, the linker domain undergoes regulatory phosphorylation by JNK and cyclin-dependent kinase (CDK) pathways [31].

Activated Smad2/3 complex forms hetero-oligomers with Smad4. In association with Smad4, the Smad2/3 complex is translocated to the nucleus, where it initiates transcription of TGF-*β*1 target genes. This pathway is regulated by several autoinhibitory feedback loops, for example, Smad7, Ski, SnoN, and Bambi are negative regulators of TGF-*β*1 signaling [22, 32].

Studies of genetically altered mice have demonstrated the importance of TGF-*β*1 signaling pathway for development of fibrosis. Overexpression of TGF-*β*1 in transgenic mice results in fibrosis of multiple organs [1], and TGF-*β*1^−/−^ mice strongly attenuate the development of liver fibrosis [1, 33, 34]. Consistently, Smad3^−/−^ mice, which lack the Smad3 signaling molecule downstream of TGF-*β*1, are much less susceptible to liver fibrosis than wild type mice [24, 25, 35]. The role of Smad2 in fibrosis is less well characterized due to the lethal phenotype of Smad2^−/−^ mice, but in comparison with Smad3, seems to regulate a distinct set of target genes [36]. TGF-*β* signaling can also be mediated by noncanonical, “non-Smad,” signaling pathways, triggered by phosphorylation of the Smad linker region [37] or by recruitment of other proteins, such as MAPK, PP2A/p70^S6K^, RhoA, and TAK1/MEKK1 to the activated TGF*β* receptor complex without a direct effect on Smad activation [37, 38].

### 2.2. NF*κ*B

Nuclear factor *κ*B (NF*κ*B) is a key transcription factor involved in a broad range of biological processes, including immune responses, cell survival, stress responses, and maturation of various cell types [39]. NF*κ*B is composed of hetero- or homodimers formed by the Rel protein family (p65, p50, p52, c-Rel, and RelB), all containing the Rel homology domain (RHD) [40, 41]. The canonical p65 : p50 heterodimer is the most abundant dimer in NF*κ*B signaling pathway [40, 41]. Generation of each monomer is transcriptionally regulated, but p50 and p52 are also regulated by processing of precursor proteins p105 and p100, respectively [39].

**Figure 2:**
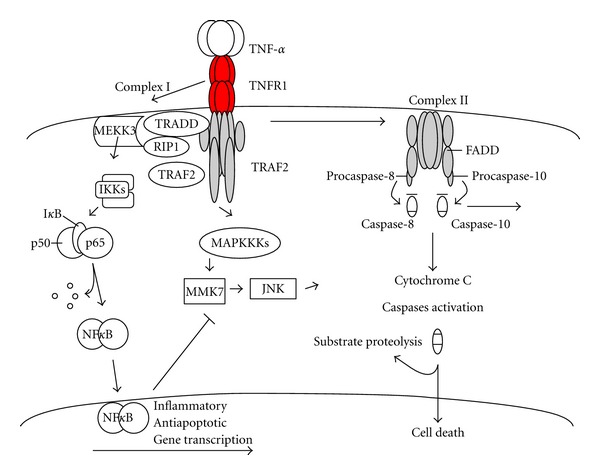
Formation of the NF*κ*B-stimulating TNFR1 signaling complex. The classical NF*κ*B pathway is activated by a broad range of stimuli, including TNF-*α*. Binding of TNF-*α* to TNFR1 triggers recruitment of the death domain-containing proteins RIP1 and TRADD. Next, complex TRAF2-cIAP1/2 is recruited to TNFR1-bound TRADD. Recruitment of IKK2 subunit to TRADD-bound TRAF2 stimulates kinase activity of the IKK complex, following by proteolytic degradation of I*κ*B proteins. p65/p50 complex (NF*κ*B) is translocated to the nucleus to activate transcription of target genes. Alternative NF*κ*B pathway (not shown) is activated by a limited subgroup of TNF ligands and involves activation of NIK-mediated stimulation of IKK1 and conversion of p100-containing NF*κ*B complexes into p52-containing NF*κ*B complexes by proteolytic processing of p100 to p52. In addition, in TNF-mediated apoptosis, receptor aggregation results in recruitment of the adaptor protein Fas-associated death domain (FADD/MORT1) and caspase-8. Caspase-8 becomes activated and initiates apoptosis by direct cleavage of downstream effector caspases. The mitochondrial pathway is initiated by the release of apoptogenic factors such as cytochrome *c*, or Smac from mitochondria into the cytosol, which trigger caspase-3 activation through the formation of the cytochrome *c*/Apaf-1/caspase-9-containing apoptosome complex.

The functional activity of NF*κ*B is determined by its natural stoichiometric inhibitors I*κ*Bs [40, 41]. The classical inhibitor proteins in the NF*κ*B signaling system consist of the single polypeptide I*κ*Bs: I*κ*B*α*, I*κ*B*β*, and I*κ*B*ε*, characterized by their ankyrin repeat domain (ARD) [40, 41]. In resting cells, I*κ*B binds the NF*κ*B dimer and prevents its nuclear translocation and DNA binding. I*κ*B*α* is the most common inhibitor, which directly interacts with NF*κ*B to form inactive complexes residing in the cytoplasm. Following cytokine stimulation, activation of the I*κ*B kinase (IKK) in turn induces phosphorylation, ubiquitination and subsequent I*κ*B*α* degradation, releasing active NF*κ*B [42] (Figure 2). Released NF*κ*B translocates to the nucleus where it initiates transcription of NF*κ*B target genes via direct binding to NF*κ*B-specific DNA motifs (GGGRNNYYCC, where R is purine, Y is pyrimidine, and N is any base). Interestingly, I*κ*B*α* itself is one of the NF*κ*B target genes [40, 41]. Synthesis of other members of the I*κ*B family is also dependent on NF*κ*B activity via negative feedback. Subsequent studies have suggested that there are two pathways of NF*κ*B activation [39]. The canonical NF*κ*B pathway is defined as being mediated by a NEMO-dependent kinase IKK (IKK*γ*) activation; while the noncanonical pathway is NEMO-independent kinase complex involving IKK*α* (IKK1) and the NF*κ*B-inducing kinase (NIK) [43]. In the canonical pathway, preexisting, latent NF*κ*B dimers are released from classical I*κ*Bs. In the noncanonical pathway, new synthesis of p100 and RelB allows for generation of RelB : p52 which is insensitive to I*κ*B control and thus translocates to the nucleus [39].

The importance of these findings has been confirmed using knockout mice. Thus, deletion of NEMO (IKK*γ*) resulted in embryonic lethality in mice caused by a massive apoptosis in the fetal liver [44]. Similar to that, the IKK*β* (IKK2) knockout [45] and the RelA knockout [46] have a lethal phenotype, suggesting that all these proteins are involved in one signaling axis of NEMO-IKK*β*-RelA. IKK*α* may compensate for the loss of IKK*β* (IKK2) [47]. Moreover, studies of genetically deficient mice demonstrate an essential role of the noncanonical NF*κ*B pathway in various biological processes, including regulation of developmental signals. Thus, mice lacking *RelB* ^−/−^, *NfκB*2^−/−^, and *NIK* ^−/−^ have defective development of lymph nodes and Peyer's Patch [39, 48, 49].

### 2.3. LPS-TLR4

**Figure 3:**
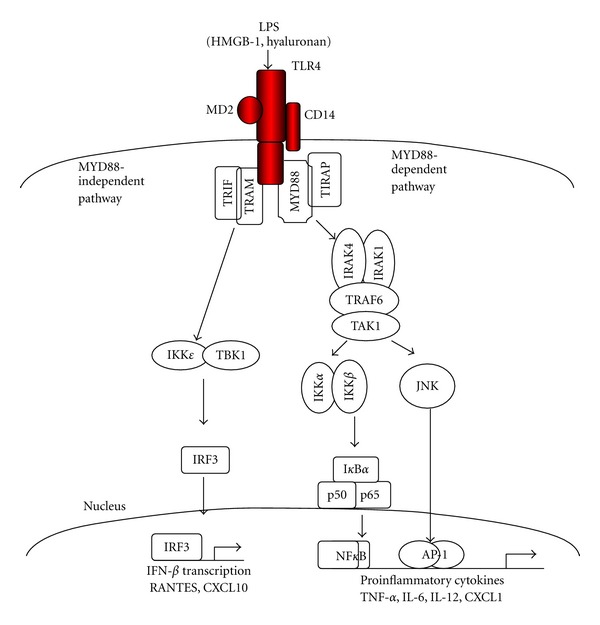
Schematic overview of TLR4 signaling pathways. LPS and other ligands bind TLR4, which transmit the signals through MyD88 to the activation of NF*κ*B and p38/c-Jun N-terminal kinase (JNK). TIRAP bridges TLR4 with MyD88. TRAF is utilized by TLR4/TRAM to activate TBK1/inhibitor of NF*κ*B kinase (IKK)*ϵ* leading to IRF-3 activation followed by IFN-*β* production.

Liver fibrogenesis is associated with increased intestinal permeability [1]. Bacterial products, including lipopolysaccharide (LPS, cell walls of gram-negative bacteria), signal via Toll-like receptor pathways. Toll-like receptors (TLRs) are innate immune signal receptors which recognize pathogen-associated molecular patterns (PAMP) such as LPS, peptidoglycan, and bacterial derived unmethylated CpG-DNA. In addition, endogenous ligands (alarmins) can bind TLR4 in the presence of CD14 and LPS binding protein (LBP) and transduce similar signals [50]. Thus, endogenous ligand HMGB-1, hyaluronan, and products of dying cells have been shown to trigger TLR signaling. LPS binds to TLR4 with its coreceptors MD-2 and CD14 and transmits its signal through adaptor proteins MyD88, TIRAP, TRIF, and TRAM to activate the kinases, IRAK1, IRAK4, TAK1, JNK, and IKK (Figure 3). These intracellular kinases lead to the activation of the transcription factors NF*κ*B, AP-1, and interferon regulatory factors (IRFs), resulting in the induction of potent innate immune responses [3, 51]. Upon activation of TLRs, cells produce proinflammatory cytokines, such as TNF-*α*, IL-6, IL-1, MCP-1, and RANTES [50].

Using TLR4-chimeric mice and *in vivo* lipopolysaccharide (LPS) challenge, Seki et al. have shown that quiescent hepatic stellate cells (HSCs), the main precursors for myofibroblasts in the liver, are the predominant target through which TLR4 ligands promote fibrogenesis. In quiescent HSCs, TLR4 activation not only upregulates chemokine secretion and induces chemotaxis of Kupffer cells, but also downregulates the transforming growth factor TGF-*β*1 pseudoreceptor Bambi to sensitize HSCs to TGF-beta-induced signals and allow for unrestricted activation by Kupffer cells [3].

TLRs are critical in liver fibrosis [3, 52]. Patients with hepatic cirrhosis have elevated portal vein levels of LPS. Portal hypertension can damage the intestinal mucosa compromising its barrier function and trigger bacterial translocation. The liver has a unique vascular system within the gastrointestinal tract, as the majority of the liver's blood supply comes from the intestine through the portal vein. When the intestinal barrier function is disrupted, an increase in intestinal permeability leads to the translocation of intestine-derived bacterial products such as lipopolysaccharide (LPS) and unmethylated CpG containing DNA to the liver via the portal vein. These gut-derived bacterial products stimulate innate immune receptors, namely Toll-like receptors (TLRs), in the liver. TLRs are expressed on Kupffer cells, endothelial cells, dendritic cells, biliary epithelial cells, hepatic stellate cells, and hepatocytes. TLRs activate these cells to contribute to acute and chronic liver diseases [53–56]. Therefore, LPS derived from the intestinal microflora is a strong candidate for the TLR4 ligand in hepatic fibrosis [57]. Consistently, gut sterilization with antibiotics attenuates liver fibrosis, and pathogen free animals are resistant to liver fibrosis [3]. Mice with deficiencies in components of TLR4 signaling pathway, CD14, LPS binding protein (LBP), or TLR4 have impaired TLR signaling and are less susceptible to liver fibrosis [58].

In addition, fragments of fibronectin have been implicated in physiological and pathological processes, especially tissue remodeling associated with inflammation [17]. Cellular fibronectin containing an alternatively spliced exon encoding type III repeat extra domain A (EDA) is produced in response to tissue injury [59, 60]. EDA-containing fibronectin fragments produce cellular responses similar to those provoked by bacterial lipopolysaccharide (LPS). EDA-containing fibronectin binds to and activates TLR4 [60], inducing nuclear translocation of nuclear factor NF*κ*B [61].

### 2.4. Stat3 Signaling

The Janus kinase-signal transducers and activators of transcription (Jak-Stat) signaling pathways are activated in the liver by more than 20 cytokines and growth factors and play a critical role in a variety of cellular functions, such as antiviral defense, acute phase response, hepatic injury, repair, inflammation, transformation, and hepatitis [62, 63]. Stat3 was originally identified as an acute-phase response factor, activated by IL-6 and other cytokines [64], but has been implicated in cellular transformation and carcinogenesis [65]. Stat3 is expressed in most tissues and early during postimplantation. Consistent with this, disruption of the Stat3 gene leads to an early embryonic lethal phenotype [66]. Tissue specific Stat3 knockouts have been generated using floxed alleles. Stat3 deficient T cells exhibit a lower proliferative response to IL-6, which suppresses apoptosis in these cells [67]. Stat3 deleted macrophages (and neutrophils) have aberrant IL-10 signaling and immune regulation [68]. Stat3 null mammary gland epithelial cells exhibit a delay in programmed cell death that occurs during cyclical gland involution [69]. Mice with Stat3-deficient hepatocytes exhibit defects in their ability to induce acute phase response genes (e.g., serum amyloid protein (SAP), fibrinogen (FB), haptoglobin (HP), serum amyloid A protein (SAA), and hemopexin (Hpx) in response to IL-6 [70]. In addition, Stat3 signaling in hepatocytes provides antiapoptotic cytoprotection [71]. Deletion of this pathway abolishes the induction of the acute-phase response, leading to more severe cholestasis and an enhanced inflammatory response with increased TNF-*α* expression and subsequent cytotoxicity [71].

**Figure 4:**
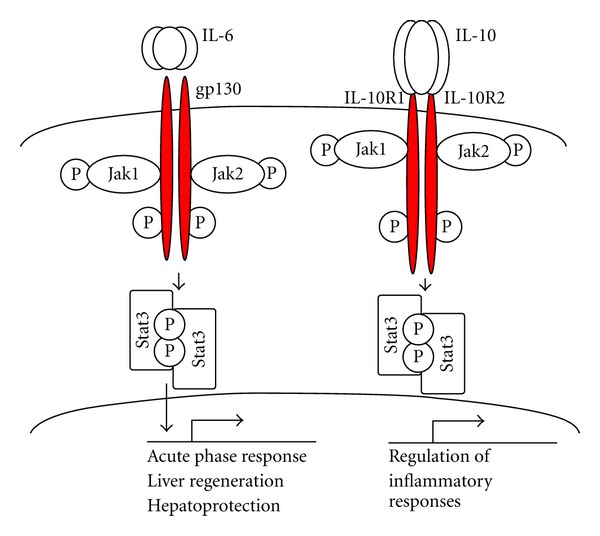
Schematic overview of Jak/Stat signaling pathways. IL-6 signals through gp130, which is a common receptor chain for IL-6 and the IL-6 receptor. Hepatocytes express high level of gp130 and IL-6. IL-6 binding to its corresponding receptors leads to the dimerization of gp130, followed by dimerization of gp130-associated Jak 1 and Jak2, and phosphorylation of Jaks and gp130. This receptor-kinase complex then recruits and phosphorylates cytoplasmic protein Stat3. Phosphorylated Stat3 forms dimers, translocates into the nuclei, and induces gene transcription. Binding of IL-10 to its corresponding receptors IL-10R1 and IL-10R2 leads to Jak and then Stat3 phosphorylation, which then functions as a transcription factor.

Cytokine signaling plays a pivotal role in the pathogenesis of liver fibrosis, which was assumed to be linked to deregulation of Th1/Th2 homeostasis towards Th2 responses [72]. However, expression of profibrogenic cytokines does not always correlate with the Th1/Th2 classification. Thus, despite driving a Th2 response, IL-6 and IL-10 have antifibrogenic effects (Figure 4). Hepatic fibrosis was increased in IL-6^−/−^ mice and in IL-10^−/−^ mice due to the loss of hepatocyte protection [73–75]. IL-22, a member of the IL-10 family of cytokines, also signals via the Jak2-Stat3 pathway and mediates hepatocyte survival during liver injury [76, 77].

## 3. Signaling Cascades Activated in Different Cell Types During Liver Fibrogenesis

### 3.1. Hepatocytes

Hepatocytes contribute to 80% of liver mass. Hepatocytes play a critical role in metabolism and detoxification for the organism [78] and are the major storage of glycogen. In the normal adult liver, mature hepatocytes exhibit a quiescent phenotype, stay in the G0 phase of the cell cycle, and show minimal turnover. However, upon hepatocyte loss (such as toxic liver injury, infection, or surgical resection), these mature hepatocytes proliferate, while maintaining their metabolic function. Hepatocyte function is heterogeneous, in part due to their location within the acinus [79, 80]. For example, while pericentral hepatocytes (adjacent to the central vein) express glutamine synthase, ornithine aminotransferase, and thyroid hormone receptor *β*1, periportal hepatocytes (adjacent to the portal triad) upregulate HNF-*α* and convert ammonia to urea [81, 82].

#### 3.1.1. TGF-*β*-Smad2/3 Signaling

TGF-*β* signaling in hepatocytes is implicated in negative regulation of the growth response. TGF-*β* type I receptor (T*β*RI) differentially phosphorylates COOH-tail serine residues of receptor-activated Smad (R-Smad, which include Smad2 and Smad3 to create three phosphorylated forms (phosphoisoforms) [30]: COOH-terminally phosphorylated R-Smad (pSmad2C and pSmad3C), linker-phosphorylated R-Smad (pSmad2L and pSmad3L), and dually phosphorylated R-Smad (pSmad2L/C and pSmad3L/C) [9, 83, 84]. While pSmad2L resides in the cytoplasm, the other phosphoisoforms are localized to cell nuclei [31]. In homeostasis, TGF-*β*-mediated pSmad3C signaling in hepatocytes opposes proliferative responses induced by mitogenic signals, causing arrest of cell cycle progression in the G1 phase by downregulation of c-Myc and induction of p21^WAF1^ [28, 85]. Acute liver injury induces secretion of proinflammatory cytokines, as well as TGF-*β* and activin A [9]. In turn, the loss of liver parenchyma triggers proliferation of resting hepatocytes. Even though TGF-*β* and activin concentrations are elevated, mitogenic proinflammatory cytokines regulate liver regeneration by shifting from cytostatic pSmad3C signaling to mitogenic pSmad3L signaling. This phenomenon allows hepatocytes to acquire “temporary resistance” to TGF-*β* (and activin A) and proliferate during liver regeneration [86, 87]. Inflammatory cytokine-induced pSmad3L stimulates c-Myc transcription [88, 89], which increases the proliferation of regeneration of hepatocytes and suppresses the cytostatic action of the pSmad3C/p21^WAF1^ pathway [9].

TGF-*β*-Smad2/3 signaling in hepatocytes has also been implicated in epithelia-to-mesenchymal transition (EMT), a process in which fully differentiated epithelial cells undergo phenotypic transition to fully differentiated mesenchymal cells (fibroblasts or myofibroblasts) [90]. During EMT, epithelial cells detach from the epithelial layer, lose their polarity, downregulate epithelial markers (e.g., hepatocyte marker albumin, cytokeratins, cadherins) and tight junction proteins (zonula occludens-1, ZO-1), increase their motility, and obtain a myofibroblast phenotype [91], with upregulated expression of *α*-smooth muscle actin (SMA) and vimentin in EMT-originated myofibroblasts. Epithelial cells transitioning into myofibroblasts are also reported to express fibroblast specific protein-1 (FSP1, S100A4), which is used as a marker of EMT in fibrogenesis and cancer [91–94]. Hepatocytes have been implicated in EMT in response to liver injury [95], suggesting that mature hepatic epithelial cells can contribute to fibrogenic myofibroblasts and collagen production in response to injury. EMT has been originally described during embryonic development [91] and plays a critical role in TGF-*β*-induced organogenesis. However, the role of EMT in fibrogenic disease has been recently questioned [96]. Studies based on the genetic labeling of hepatocytes, using Albumin-Cre mice, have demonstrated that hepatocytes are capable of differentiating into myofibroblasts *in vitro*, but not *in vivo *[97]. Moreover, FSP1 is not a robust marker for EMT, since its expression is not restricted to EMT-transitioning cells, but is expressed by myelomonocyic lineage cells [98–100].

TGF-*β* induces expression of growth factors and cytokines by hepatocytes. Moreover, hepatocytes serve as a significant source of BMP-7, a natural inhibitor of the TGF-*β*1-signaling pathway belonging to the TGF-*β* superfamily. Administration of BMP-7 in pharmacological doses attenuates the development of kidney fibrosis and liver fibrosis [101–103]. Hepatocyte-specific deletion of Smad7 results in spontaneous liver dysfunction and aggravation of alcohol-induced liver injury [104].

#### 3.1.2. LPS-TLR4 Signaling

Consistent with their filtering/detoxification function, hepatocytes express TLRs which are constitutively engaged by bacterial products in the liver [105]. Primary cultured hepatocytes express mRNA for all TLRs, but are capable of responding only to TLR2 and TLR4 ligands *in vitro*. However, TLR2 and TLR4 signaling in hepatocytes is fairly weak *in vivo* [106–108]. Under inflammatory conditions, hepatocytes upregulate TLR2 and become more sensible to TLR2-mediated signals. At the same time, TLR4 expression in hepatocytes is not strongly upregulated [109]. Although hepatocytes express TLR4 and are capable of transmitting TLR4 signals *in vitro*, the contribution of TLR4 signaling in hepatocytes is limited. Meanwhile, the TLR/MyD88-mediated pathway is required for the initiation of liver regeneration after partial hepatectomy (PH) [108, 110].

#### 3.1.3. TNF-*α*-NF*κ*B

Chronic injury causes an imbalance between the production of protective and damaging cytokines, resulting in the activation of apoptotic signals in hepatocytes. TNF-*α* and related cytokines play a key role in mediating hepatocyte homeostasis by regulating both anti- and proapoptotic pathways. TNF-*α* signals through TNF-R1 and TNF-R2, of which TNF-R1 plays a critical role in TNF-*α*-mediated activity in the liver. The proapoptotic effects of TNF-*α* result from a cascade of caspase activation. This pathway is initiated by TNF-*α*-induced TNF-R1 receptor crosslinking, recruitment of TRADD and FADD (adaptor protein TNF receptor TRADD and Fas-associated death domain FADD) and cleavage of caspase 8, which activates the downstream proapoptotic caspases (caspases 3, 6, 7). In turn, the activation of TNF-*α*-dependent prosurvival signals is mediated by NF*κ*B activation and involves transcriptional expression of suppressors of apoptosis, including Bcl-2, Bcl-xL, and Bfl-1 [111]. However, TNF*α* also activates NF*κ*B, rendering hepatocytes resistant to apoptosis unless also treated with cycloheximide, actinomycin D or the super-repressor of I*κ*B [112, 113].

#### 3.1.4. IL-6-Stat3 Signaling

In hepatocytes, IL-6 plays a crucial role in liver regeneration and transmits its mitotic signals mainly through Stat3. IL-6 stimulates hepatocytes to produce acute-phase response proteins, including serum amyloid A, complement C3 and C-reactive protein. In IL-6-deficient mice, Stat3 activation is dramatically suppressed in hepatocytes [80]. Although Stat3 signaling can be induced by other cytokines, such as G-CSF [114] and leptin [115], current data suggests that Stat3 in hepatocytes is almost exclusively activated by IL-6. Thus, hepatocyte regeneration in response to partial hepatectomy triggers activation of the IL-6/Stat3 signaling pathway, composed of IL-6 receptor, gp130, receptor-associated Janus kinase (Jak), and Stat3. The IL-6 receptor forms a complex with two molecules of gp130 [62].

Stat3 promotes liver regeneration by promoting cell cycle progression from G1 to S phase [116]. Stat3 regulates the expression of cyclin D1 [117], which is required for hepatocyte proliferation [118]. As expected, hepatocyte-specific Stat3-deficient mice exhibit impaired DNA synthesis and mitotic activity of hepatocytes after partial hepatectomy.

Other target genes of Stat3 include antiapoptotic genes *FLIP*, *Bcl*-*2*, and *Bcl*-*xL* [118, 119]. Therefore, it is believed that Stat3 prevents liver damage by its antiapoptotic and promitogenic effects [78]. Deletion of the gp130/Stat3 pathway in hepatocytes leads to increased hepatotoxicity and accelerates liver injury and inflammation [71]. This effect is likely mediated via the Stat3 induction of EGFR and IGF-1 signaling pathways [120]. Interestingly, Stat3 also possesses antioxidative capacity. Hypoxia and reperfusion injures hepatocytes via the generation of reactive oxygen species (ROS) and activation of redox-sensitive caspases such as caspase-3/-9. Stat3 upregulates Ref-1 [121] and Mn-SOD [122], which protects hepatocytes from ROS-mediated apoptotic cell death [118]. Thus, activation of Stat3 in hepatocytes has hepatoprotective and anti-fibrotic effects [123].

### 3.2. Kupffer Cells

Kupffer cells are liver-resident macrophages, which are long lived and radiation resistant. They express myeloid markers such as F4/80, CD68, CD11b, CCR2, and CX3CR1 [51, 124, 125]. However, there are currently no specific markers distinguishing Kupffer cells from recruited BM derived macrophages. It is believed that both of these populations actively participate in development of liver fibrosis by secretion of TGF-*β*1, IL-6 and other profibrogenic cytokines.

#### 3.2.1. TLR Signaling

Kupffer cells are well established targets for the TLR4 ligand LPS and produce inflammatory and fibrogenic cytokines, which may activate HSCs [126]. However, TLR4 signaling in Kupffer cells is not critical for the pathogenesis of experimental liver fibrosis. Deletion of TLR4 signaling in BM-derived inflammatory cells and Kupffer cells was achieved in BM-chimeric mice, pretreated with clodronate (to reconstitute long-lived Kupffer cells). Interestingly, only a modest inhibition of liver fibrosis was observed in these mice in response to liver injury [3, 51]. However, LPS, but not TGF-*β*, is a strong activator of Kupffer cells *in vitro* and *in vivo*. LPS-stimulated Kupffer cells secrete TNF-*α* and TGF-*β*. Furthermore, experimental alcoholic liver disease requires TLR4 on BM derived macrophage and Kupffer cells [51, 127]. Interestingly, only a modest inhibition of liver fibrosis was observed in these mice in response to liver injury. These results indicate that LPS-induced fibrosis does not need Kupffer cell-mediated activation of HSC.

#### 3.2.2. IL-6-IL-10-Stat3 Signaling

IL-6 and IL-10 induce opposing effects on macrophages. IL-6 signals via gp130 and IL-6R and promotes inflammatory responses in Kupffer cells/macrophages. In turn, IL-10 secreted by Th1 and T cells stimulate IL-10R1 and IL-10R2 on Kupffer cells/macrophages, causing their prolonged activation. Activation of IL-10-Stat3 signaling inhibits inflammatory responses. Stat3 upregulates expression of the suppressor of cytokine signaling 3 (SOCS3) [128], which binds to gp130, limiting IL-6-induced inflammatory responses [78]. Thus, while IL-6 triggers proinflammatory responses in macrophages, IL-10 mediates anti-inflammatory responses that are associated with decreased liver fibrosis [78, 128].

### 3.3. Endothelial Cells

Liver sinusoidal endothelial cells (LSECs) maintain the integrity of hepatic sinusoids and mediate barrier function, blood clearance, vascular tone, immunity, hepatocyte growth, and injury-induced angiogenesis [129–131]. LSECs differ from other ECs by the lack of basement membrane and together with hepatic stellate cells HSCs (residing in the space of Disse and acting as pericytes in the normal liver), form a fenestrated monolayer which regulates the blood supply to underlying hepatocytes [1]. Regulation of hepatic vascular tone is mediated by HSC contractility mediated by endothelin-1, angiotensin II, norepinephrine, prostaglandin F2, thromboxane A2, and thrombin [1, 132]. Disruption of the integrity of the endothelium results in defenestration and capillarization of LSECs, and activation of an antifibrinolytic cascade to support coagulation and hemostasis [133]. In turn, LSECs secrete cytokines and soluble factors (such as monocyte chemoattractant protein 1 (MCP-1) and endothelin-1(ET-1)) that induce recruitment of inflammatory cells, contractility of HSCs, and platelet aggregation and degranulation [129]. In turn, as a part of a wound healing process, LSECs proliferate and migrate. In response to chronic injury, numerous mediators of angiogenesis, including angiopoietins, transforming growth factor (TGF-*β*1), platelet-derived growth factor (PDGF), tumor necrosis factor-alpha (TNF-*α*), interleukins, and members of the fibroblast growth factor family (FGF), are produced [134]. However, vascular endothelial growth factor (VEGF) remains the strongest inducer of angiogenesis [135].

Damage to the hepatic endothelium is further increased by portal hypertention and NO production, which accompany fibrogenic liver injury. NO production increases vasodilation and permeability of LSECs. Dysfunctional LSECs contribute to local production of NO production, further facilitating liver injury [10, 133].

#### 3.3.1. Angiotensin 1 and VEGF

VEGF is an important regulator of angiogenesis and vascular tone. VEGF controls LSEC survival, proliferation, migration, and angiogenesis. VEGF binds to the receptor VEGFR2 and mediates its biological responses through reactive oxygen species (ROS) [136]. Another strong angiogenic factor, Angiopoietin 1 (Ang 1), regulates maturation and stability of blood vessels. Moreover, neovascularization induced during the development of livers fibrosis is mediated by Angiopoietin 1, expressed mostly by activated HSCs [137]. In turn, Ang 1 signals through endothelial receptor tyrosine kinase Tie2 and synergistically enhances VEGF's effects [138]. VEGF binds to its receptor and activates an Akt signaling cascade to increase vascular tone and the formation of collateral circulation [139, 140].

Furthermore, proliferation and migration of endothelial cells depends on pericyte coverage of vascular sprouts for vessel stabilization. This process is regulated by VEGF and platelet-derived growth factor (PDGF) through their cognate receptors [141]. VEGFR is expressed on endothelial cells, and PDGFR is expressed on HSCs and vascular smooth muscle cells (VSMCs). Moreover, it is believed that PDGF-R*β* is exclusively expressed by HSCs in the liver and strongly upregulated in HSCs in response to fibrogenic liver injury [1]. PDGF induces neovascularization by priming VSMCs/pericytes to release pro-angiogenic mediators. VEGF acts as a negative regulator of neovascularisation. Specifically, while pericyte-derived PDGF mediates angiogenesis, VEGF ablates pericyte coverage of nascent vascular sprouts, leading to vessel destabilization. VEGF-mediated activation of VEGF-R2 suppresses PDGF-R*β* signaling in HSCs/pericytes/VSMCs through the assembly of a previously undescribed receptor complex consisting of PDGF-R*β* and VEGF-R2 [141]. Thus, VEGF ameliorates development of liver fibrosis and can serve as a novel target for anti-fibrotic therapy [142].

#### 3.3.2. TGF-*β*-Signaling

TGF-*β* signaling in endothelial cells plays a critical role in vascular development and maintenance of vascular homeostasis. Mice deficient for various TGF-*β* signaling components have an embryonic lethal phenotype due to vascular defects, abnormal yolk sac vasculogenesis and/or angiogenesis [143, 144]. TGF-*β* is also essential for vascular integrity in the adults due to its role in regulation of anti-inflammatory characteristics of endothelial cells, growth and migration [145]. Similar to other cell types, TGF-*β* signaling in endothelial cells results in activation of TGF-*β* receptors with Ser/Thr kinase activity. The effect of TGF-*β* on endothelial cells is dose-dependent. Low levels of TGF-*β* promote angiogenesis, while higher doses inhibit angiogenesis [146]. TGF-*β* regulates the activation of the endothelium via two opposing type I receptor/Smad pathways, activin receptor-like kinase (ALK)1 and ALK5 [145]. The classical TGF-*β* type I receptor that activates Smad2/3 signaling is ALK5 (TGF*β*-RI). ALK2 (ActRI) is typically used by BMPs to activate Smads1/5/8. Use of ALK2 by TGF-*β* is rather an exceptional nonhepatic event [147]. Typically the Smad2/3 pathway is activated by the type I receptors ALK4, 5 or 7 [28]. Furthermore, a coreceptor of TGF-*β*, endoglin (CD105), is upregulated on proliferating endothelial cells and facilitates effective TGF-*β*-ALK1 signaling [148, 149]. Another molecule which regulates TGF-*β* signaling is VE-cadherin. VE-cadherin-deficient endothelial cells demonstrate a loss of TGF-*β*-induced inhibition of endothelial cell proliferation and motility [145, 150].

TGF-*β* signaling in endothelial cells may contribute to fibrosis via transition to mesenchymal cells (EndMT), giving rise to myofibroblasts in response to fibrogenic injury. EndMT has been reported to contribute to cardiac [151] and renal [152] fibrosis. The generation of mesenchymal profibrotic cells from endothelial cells by this process appears to recapitulate the transdifferentiation of endothelial cells that leads to the formation of the cardiac valves in embryonic development [153]. EndMT is identified by expression of myofibroblasts-like genes [91] in endothelial cells that are expressing or have a “history” of expressing PECAM-1/CD31, Tie-1 [151], Tie-2 and CD34 [152, 154]. A difficulty in interpreting these studies is that it is now recognized that Tie-2 is not a specific marker for endothelial cells in that it is also expressed in BM derived hematopoietic cells. Although endothelial cell injury [10] and neovascularization play a critical role in liver fibrosis, the role of EndMT in liver fibrosis is unknown.

#### 3.3.3. TLR4-Signaling

LSEC are exposed to endogenous LPS liver injury. LPS induces upregulation of TLR4 expression in LSECs to facilitate angiogenesis [131]. *In vitro*, this effect is dependent on Myd88 activation and is associated with secretion of MMP2 by LSEC. *In vivo* studies have supported this data, demonstrating that TLR4-deficient mice exhibit attenuated angiogenesis and fibrosis [155].

Low, physiological concentrations of endotoxin are continuously present in portal venous blood, and the liver mediates intrinsic signals to develop tolerance [155]. LPS induces the release of IL-10 from LSECs and Kupffer cells and also downregulates CD4^+^ T cell activation by LSECs through down-modulation of the expression of MHC class II, CD80 and CD86. In contrast, TLR4 activation of professional APC by endotoxin increases T cell activation. These observations explain why the tolerogenic effect in the liver seems to be related to the continuous exposure of sinusoidal cells to bacterial products from the gut (reviewed in [155]). Following initial activation of LSECs, Kupffer cells are a tolerogenic cellular population contributing to the tolerogenic properties within the liver [155].

#### 3.3.4. TNF-*α*-NF*κ*B

In response to liver injury, release of endogenous LPS mediates release of TNF-*α*, which in turn triggers expression of target genes in LSECs. However, a specific role of NF*κ*B in LSECs in the pathogenesis of liver fibrosis has not been evaluated [156]. Experiments in transgenic mice overexpressing the IkB-*α* super-repressor in endothelial cells, have demonstrated that inhibition of the NF*κ*B signaling pathway in LPS-stimulated mice causes a defect in expression of endothelial tight junction proteins, and as a result, a loss of integrity of the endothelium and increased vascular permeability [157], suggesting that NF*κ*B is responsible for the stress-induced responses of the endothelium to septicemia or TNF-*α*.

#### 3.3.5. Stat3

The role of Stat3 in endothelial cells has not been carefully studied. It has been suggested that Stat3 facilitates protection of endothelial cells (LSEC) exposed to endogenous LPS liver injury and inflammation. IL-6 has a protective effect on hepatic LSECs by activation of Stat3 signaling [158–160]. Consistent with this, endothelial-specific Stat3-deficient mice are more susceptible to alcohol-induced injury, demonstrating a critical function of the endothelium and LSECs in chronic liver injury [160]. Recent study has implicated Stat3 signaling in endothelial cells in mediating dual anti-inflammatory and antiapoptotic functions, of attenuating hepatic inflammation and SEC death during alcoholic liver injury [161].

### 3.4. Cholangiocytes

Cholangiocytes, the epithelial cells lining intrahepatic bile ducts, are ciliated cells. Each cholangiocyte has a primary cilium consisting of a microtubule-based axoneme and the basal body, centriole-derived, microtubule-organizing center from which the axoneme emerges. Cholangiocyte cilia extend from the apical plasma membrane into the bile duct lumen [162]. Cholangiocytes, the biliary epithelial cells, can be identified by their apical structure and expression of specific keratins, such as K-19 [163]. Cholangiocytes originate from the common epithelial progenitor in the liver during development. Unlike hepatocytes, they lack the ability to regenerate their mass [164]. However, cholangiocytes are capable of proliferation in response to cholestatic liver injury, and this phenomenon has been referred as the ductular reaction [1]. It is believed that cholangiocytes participate in the activation of portal fibroblasts, located in close proximity. Cholangiocytes have been implicated in secretion of a variety of cytokines and factors, which accelerate development of liver fibrosis [165], including NGF, MCP-1 growth factors HGF, VEGF, CTGF, and endothelin-1 [166]. However, it remains unclear if cholangiocytes serve as a significant source of cytokines.

#### 3.4.1. TGF-*β*-Smad

Difficulties associated with the isolation and culturing of a pure population of cholangiocytes is a limiting factor in investigating the role of cholangiocytes in fibrogenic liver injury. It has been suggested that similar to hepatocytes, cholangiocytes are capable of differentiation into fibrogenic myofibroblasts via epithelial-to-mesenchymal transition (EMT) in response to TGF-*β*-induced liver injury [167, 168]. Although EMT in hepatocytes has been documented *in vitro*, *in vivo* studies in adult mice using Cre-lox-based cell fate mapping have not confirmed this finding [97]. Similarly, genetic labeling of K19^+^ cholangiocytes has demonstrated that cholangiocytes do not contribute to fibrogenic myofibroblasts in experimental cholestatic liver injury [163, 169]. Moreover, hepatic epithelial cells and their precursors, genetically labeled in alpha-fetoprotein-Cre mice, do not differentiate into fibrogenic myofibroblasts in adult mice [96, 170].

#### 3.4.2. TLR Signaling

A few studies have mostly linked TLR signaling in cholangiocytes to anti-microbial immunity [171, 172]. Cholangiocytes may participate in microbe-associated, hepatic proinflammatory responses. *In vitro* studies of cultured human cholangiocytes suggest that LPS-TLR-signaling pathway activate the small GTPase Ras that mediates cholangiocyte proinflammatory cytokine production and proliferation [172].

#### 3.4.3. NF*κ*B and Stat3 Signaling Pathways

NF*κ*B and/or Stat3 signaling pathways in cholangiocytes have not been specifically evaluated. Meanwhile, conditional inactivation of Stat3 in hepatocytes and cholangiocytes (stat3(Deltahc) of multidrug resistance gene 2 mdr2^(−/−)^ mice strongly aggravated bile acid-induced liver injury and fibrosis [120].

### 3.5. Hepatic Stellate Cells

HSCs are perisinusoidal cells that normally reside in the space of Disse and contain retinoid lipid droplets [173, 174]. Under physiological conditions, HSCs exhibit a quiescent phenotype and express neural markers, such as glial fibrilar acid protein (GFAP), synemin, synaptophysin [1], and nerve growth factor receptor p75 [175, 176], secrete hepatocyte growth factor (HGF), and store vitamin A [177]. HSCs are also implicated in phagocytosis and antigen presentation [178, 179]. In response to injury, HSCs have decreased lipid droplets, acquire contractility, and activate into collagen type I^+^ *α*-SMA^+^ myofibroblasts. During development HSCs are derived from the translocation of submesothelial mesenchymal cells from the liver capsule [180].

#### 3.5.1. TGF-*β*-Smad2/3 Signaling

TGF-*β* signaling plays a critical role in initiating and promoting the activation of qHSCs into myofibroblasts. Nuclear localization of pSmad2 and pSmad3 is seen in the activated HSC [9]. Transgenic mice have demonstrated that overexpression of TGF-*β* produces liver fibrosis [181], and conditional induction of TGF-*β* has demonstrated that the severity of fibrosis is proportional to the level of produced TGF-*β* [24]. Smad3 is a direct mediator of matrix production in aHSCs. Mice lacking Smad3 are protected from fibrosis [25, 182]. Activation of TGF-*β* signaling causes transient expression of Smad7, regulated by a feed-back mechanism. Smad7, in turn, inhibits HSC differentiation into fibrogenic myofibroblasts and attenuates experimental fibrosis *in vivo* [183, 184]. BMP-7, another member of the TGF-*β* superfamily, also acts as a TGF-*β* antagonist and administration of BMP-7 in pharmacological doses attenuates development of kidney and liver fibrosis [101–103].

Although activation/phosphorylation of Smad2/3 is considered to be the main fibrogenic pathway in HSCs, TGF-*β*1 has been also found to mediate its profibrogenic action via an alternative ALK1/Smad1 pathway in HSCs by upregulation of Id1 (the inhibitor of differentiation (1) that facilitates HSC activation [185]. TGF-*β*1 controls the transdifferentiation process in HSCs. The recent study, aimed to elucidate TGF-*β*1 target genes responsible for fibrogenesis, has analyzed the Smad7-dependent mRNA expression profiles in HSCs, and identified that Id1 protein was strongly reduced by ectopic Smad7 expression in HSCs. In concordance, Id1 overexpression in HSCs enhanced cell activation, while knock-down of Id1 in HSCs inhibited HSC differentiation into myofibroblasts. Treatment of HSCs with TGF-*β*1 resulted in Id1 upregulation implicating Id1 to be an alternative but critical mediator of HSC activation into myofibroblasts signaling via TGF-*β*1/ALK1/Smad1 pathway [185].

Other factors can facilitate TGF-*β* signaling in HSCs. In particular, stimulation of aHSCs with platelet-derived growth factor (PDGF) and TGF-*β* produces a synergistic effect on migration and expression of MMPs [186, 187]. Moreover, PDGF promotes the activation of HSCs via activation of the PI3K-Akt signaling pathway. PI3K (phosphatidylinositol-3-kinase) activity phosphorylates PIP_2_ to generate PIP_3_ (3,4,5-trisphosphate) [188]. PIP_3_ binds to the pleckstrin homology domain of Akt, directing it to the cell membrane where it becomes activated by phosphorylation events to initiate cell survival mechanisms. Consistently, inhibition of PI3K activity suppresses cell proliferation and type I collagen gene expression in activated HSCs [189, 190]. PDGF also activates ERK in HSCs by sequential activation of Ras-Raf-MEK signaling [191] and further facilitates proliferation of aHSCs [192].

The tumor suppressor protein phosphatase and tensin homolog deleted on chromosome ten (PTEN) is a dual specificity protein and lipid phosphatase that dephosphorylates PIP_3_ [188, 193]. PTEN is a negative regulator of PI3K and ERK signaling [190]. Overexpression of PTEN attenuates collagen Type I production when aHSCs induces HSC apoptosis. Deletions of PTEN occur during malignant transformation in various tissues [193]. Decreased PTEN expression is also associated with dysregulation of tissue remodeling, such as pulmonary fibrosis, bronchial asthma, and rheumatoid arthritis [194–196]. Since the PI3K/Akt pathway stimulates proliferation and activation of HSCs, inhibiting PTEN promotes liver fibrosis [197].

#### 3.5.2. PDGF Signaling

Platelet-derived growth factor (PDGF) is a powerful mitogen for HSCs. In fibrotic liver, PDGF induces HSC proliferation, synergistically facilitating TGF-*β*1-mediated HSC activation [192]. PDGF signals through the transmembrane receptor tyrosine kinases initiating multiple signaling pathways [198, 199], including activation of the mitogen-activated protein kinase (MAPK) family implicated in cellular proliferation and transmigration. This includes the extracellular signal-regulated protein kinase (ERK) pathway and two stress-activated protein kinase (SAPK) pathways: the c-Jun N-terminal kinase (JNK) and the p38 pathway [31].

#### 3.5.3. TLR4-Signaling

**Figure 5:**
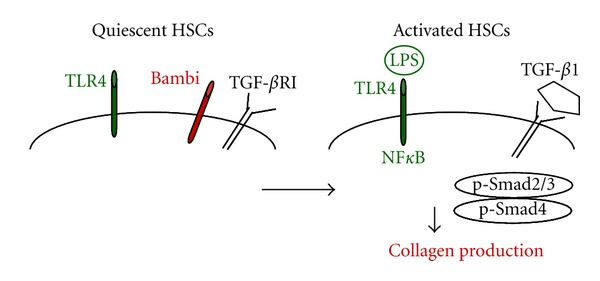
Activation of Hepatic Stellate cells following TLR4 and TGF*β* Receptor cross-talk. In aHSCS, Bambi blocks TGF*β*R activation. TLR4 activation by LPS downregulates BAMBI, so that TGF*β*1 now induces signal transductions of phosphorylated Smad2/3 and activation of collagen Type I production in HSCs.

LPS activates the NF*κ*B and JNK/AP-1 pathways in aHSCs. LPS enhances expression of the adhesion molecules ICAM-1 and VCAM-1 and TLR2 and the secretion of IL-8, MCP-1, MIP-1*α*, MIP-1*β*, RANTES, KC, MIP-2, and IP-10 in aHSCs. In turn, LPS downregulates the expression of bone morphogenetic protein (BMP) and activin membrane bound inhibitor (Bambi), a transmembrane suppressor of TGF-*β* signaling (Figure 5). Bambi is a TGF-*β* pseudoreceptor that lacks an intracellular kinase domain, and similar to activin, prevents TGF-*β* signaling. Signaling via TLR4 downregulates Bambi and facilitates TGF-*β* signaling in HSCs. Overexpression of Bambi inhibits HSC activation, while overproduction of a dominant negative form of Bambi enhances TGF-*β* signaling, and induces activation of HSCs [51].

LPS signaling is blocked by inactivation of NF*κ*B and JNK, demonstrating the role of NF*κ*B and JNK in TLR4 signaling in HSCs. TLR4 signaling in HSCs is critical for development of liver fibrosis. Bone-marrow chimeric mice with a TLR4 deficiency in recipient liver cells, including HSCs, were resistant to liver fibrosis. Since hepatocytes exhibited no response to LPS *in vivo*, HSCs were proposed to be the major cell population in the injured liver transmitting TLR4-induced fibrogenic signals [3].

#### 3.5.4. NF*κ*B

TNF-*α* has a dual role in HSC biology. TNF-*α* can produce antiapoptotic (via NF*κ*B activation), or proapoptotic (via caspase activation) and antiproliferative responses in HSC [113, 200]. The later effect is mainly attributed to the ability of TNF-*α* to regulate CD95L expression.

Activation of HSCs in response to fibrogenic liver injury is associated with increase of the basal NF*κ*B activity in comparison with qHSCs [201, 202]. NF*κ*B promotes antiapoptotic signals in aHSCs predominantly via the classic p65 : p50 complex and low levels of a p65 homodimer [200]. Increased basal activity of NF*κ*B in activated HSCs is linked to downregulation of I*κ*B-*α* [201]. Interestingly, as a result of liver injury, elevated levels of TNF-*α* further stimulate NF*κ*B activity. In turn, NF*κ*B mediates antiapoptotic functions and protects aHSCs from TNF-*α*-induced apoptosis. TNF-*α*-induced apoptosis of aHSCs can be achieved in the presence of cycloheximide, or pharmacological inhibition of IkB [200].

#### 3.5.5. Stat3 Signaling

Some Jak2-Stat3-signaling cytokines may have a direct effect on aHSCs by facilitating ECM deposition [203, 204]. Leptin increases collagen production in aHSCs/myofibroblasts in fibrotic liver [205–207] and promotes HSC survival [205]. Treatment with leptin increases the numbers of HSCs in S and G2/M phases of the cell cycle as well as increases cyclin D1 expression. Leptin mediates its function via activation of the Stat3-Jak2 signaling pathway with downstream activation of ERK, AKT and PI3K [113]. Moreover, other agonists, such as PDGF, EGF and HGF, also activate Stat3 and produce a direct profibrogenic effect on HSCs. As expected, deletion of their corresponding receptors in mice inhibits liver fibrosis [62, 63]. Taken together, there is emerging evidence that supports a significant role of Stat3 in stimulating liver fibrosis. However, the specific role of Stat3 in HSC activation using conditional ablation of Stat3 has not been investigated.

### 3.6. Portal Fibroblasts (PFs)

Portal fibroblasts are defined as spindle shaped cells of mesenchymal origin that are present in the portal tracts. Under normal conditions, they participate in physiological ECM turnover [208–211] and do not express *α*-SMA. Induced mostly by cholestatic liver injury, portal fibroblasts proliferate, though much slower than HSCs [212], and deposit collagen (e.g., type I) around the portal tracts [213].

The mechanisms of liver fibrogenesis after carbon tetrachloride (CCl_4_) or bile duct ligation (BDL) treatment are different. In the CCl_4_ model, necrosis of hepatocytes and inflammation occurs around centrolobular veins. BDL induces increased biliary pressure and moderate inflammation, causing cytokine secretion by biliary epithelial cells. PFs and HSCs are distributed differently in the hepatic lobule: HSCs resemble pericytes and are located along the sinusoids, in the space of Disse between the endothelium and the hepatocytes, whereas the portal fibroblasts are embedded in the portal tract connective tissue around portal triad [209]. Therefore, the relative activation of HSCs and PFs depend on the model of liver injury [208, 214]. Consistently, PFs have been implicated in pathogenesis of cholestatic liver injury [7, 208]. Unlike toxic liver injury, in cholestatic liver injury, PFs significantly contribute to a population of fibrogenic myofibroblasts compared to HSCs [209]. Peribiliary myofibroblasts express *α*SMA, collagen Type I, and PDGF receptor-*β* subunit. In addition, expression of IL-6 is significantly increased in peribiliary myofibroblasts in comparison with activated HSCs [210].

Very little is known about signaling in portal fibroblasts due to the inability to isolate highly purified cells for short term, primary culture. The standard method of isolation of PFs is by outgrowth of peribiliary myofibroblasts from bile duct segments [210, 215]. Differentiating PFs from other fibrogenic myofibroblasts, including aHSCs, is difficult [212]. To date, PFs are distinct from HSCs in that they express elastin (TE-7-positive antigen) and Thy-1.1 (a glycophosphatidylinositol-linked glycoprotein of the outer membrane leaflet described in fibroblasts of several organs) [216–218], do not store retinoids, and do not express desmin or neural markers [219]. Several other proteins have been suggested to be upregulated in PFs (versus HSCs), such are fibulin 1 and 2 [220], gremlin [221]. and cofilin 1 [215]. Moreover, PFs do not express cytoglobin, a protein characteristic for aHSCs [215]. In addition, compared to HSCs, PFs express different TGF-*β* isoforms which may contribute to biliary fibrosis; and distinct from HSCs, PDGF inhibits PFs proliferation and myofibroblastic differentiation [216, 222].

#### 3.6.1. TGF-*β*-Smad2/3 Signaling

It is anticipated that in response to TGF-*β* signaling, PFs upregulate collagen Type I and activate the Smad2/3 signaling pathway, similar to HSCs and other myofibroblasts [216]. Our general understanding of TGF-*β* signaling suggests that mitogenic signaling synergistically promote the growth and invasion of mesenchymal cells [84, 223]. Blocking of phosphorylation of Smad2 abrogates the synergistic responses of fibroblasts to TGF-*β* and mitogens [9, 84].

## 4. Closing Remarks

In response to chronic injury, every liver cell contributes to the pathophysiology of liver fibrosis. Several key signaling pathways have emerged that are critical for liver fibrosis. The TGF-*β*/Smad pathway has been well characterized and demonstrated to affect every liver cell type. More recent studies have demonstrated key roles for other pathways, including TLR4 and Jak/Stat3 in hepatic fibrosis. Furthermore, there is cross-talk between these fibrogenic pathways. For example, activating TLR4 signaling potentiates the TFG-*β*/Smad pathway. Although the TGF-*β* pathway might be too important in physiological homeostasis to block as a therapeutic intervention, targeting new cross-talking pathways may provide novel approaches to the treatment of liver fibrosis.

## Abbreviations

HSCs: Hepatic stellate cells
qHSCs: Quiescent HSCs
aHSCs: Activated HSCs
CCl: Carbon tetrachloride
BDL: Bile duct ligation
*α*-SMA: *α*-smooth muscle actin
ECM: Extracellular matrix
SMA: *α*-smooth muscle protein
TGF-*β*1: Transforming growth factor-*β*1
LPS: Lipopolysaccharide
LSECs: Liver sinusoidal endothelial cells.
